# The Role of CXCR3 in DSS-Induced Colitis

**DOI:** 10.1371/journal.pone.0101622

**Published:** 2014-07-03

**Authors:** Belal Chami, Amanda W. S. Yeung, Caryn van Vreden, Nicholas J. C. King, Shisan Bao

**Affiliations:** 1 Discipline of Pathology, Bosch Institute and School of Medical Sciences, Sydney Medical School, The University of Sydney, Sydney, NSW, Australia; 2 Centre for Vascular Research, Lowy Cancer Research Centre, University of New South Wales, Sydney, NSW, Australia; 3 Sydney Institute of Emerging infectious diseases and Biosecurity (SEIB), Sydney Medical School, University of Sydney, Sydney, NSW, Australia; Dasman Diabetes Institute, Kuwait

## Abstract

Inflammatory bowel disease (IBD) is a group of disorders that are characterized by chronic, uncontrolled inflammation in the intestinal mucosa. Although the aetiopathogenesis is poorly understood, it is widely believed that IBD stems from a dysregulated immune response towards otherwise harmless commensal bacteria. Chemokines induce and enhance inflammation through their involvement in cellular trafficking. Reducing or limiting the influx of these proinflammatory cells has previously been demonstrated to attenuate inflammation. CXCR3, a chemokine receptor in the CXC family that binds to CXCL9, CXCL10 and CXCL11, is strongly overexpressed in the intestinal mucosa of IBD patients. We hypothesised that CXCR3 KO mice would have impaired cellular trafficking, thereby reducing the inflammatory insult by proinflammatory cell and attenuating the course of colitis. To investigate the role of CXCR3 in the progression of colitis, the development of dextran sulfate sodium (DSS)-induced colitis was investigated in CXCR3^−/−^ mice over 9 days. This study demonstrated attenuated DSS-induced colitis in CXCR3^−/−^ mice at both the macroscopic and microscopic level. Reduced colitis correlated with lower recruitment of neutrophils (*p = *0.0018), as well as decreased production of IL-6 (*p*<0.0001), TNF (*p* = 0.0038), and IFN-γ (*p* = 0.0478). Overall, our results suggest that CXCR3 plays an important role in recruiting proinflammatory cells to the colon during colitis and that CXCR3 may be a therapeutic target to reduce the influx of proinflammatory cells in the inflamed colon.

## Introduction

Inflammatory bowel disease (IBD) is characterized by chronic, uncontrolled inflammation in the intestinal mucosa, affecting millions of people worldwide, with a corresponding economic burden [1]–[4]. Although the etiopathogenesis is poorly understood, it is believed that IBD stems from a dysregulated immune response towards otherwise harmless commensal bacteria that are normally present in the colon [5]. DSS is thought to permeablise the mucosal membrane of the colon to enteric bacterial [6] by inducing epithelial injury [7]. Interestingly, DSS may also elicit colitis by promoting the proliferation of gram-negative bacteria in mice [8].

Given the destructive nature of cellular infiltrates in the intestinal mucosa during IBD [9], [10], many studies have focused on the importance of recruitment factors, such as chemokines, in the development of the disease.

CXCR3, a chemokine receptor in the CXC family, has been implicated in the pathogenesis of several chronic diseases including rheumatoid arthritis [11], multiple sclerosis [12], dry eye syndrome [13], and psoriasis [14]. CXCR3 ligands are not constitutively expressed, and are instead induced acutely by IFN-γ on T cells, indicating an inflammatory role of CXCR3 [15]–[17]. Epithelial and endothelial cells, as well as a wide range of lymphoid cells such as memory T cells, NK cells, B cells, neutrophils and monocytes, also express CXCR3 [11], [18]–[21].

CXCR3 and its corresponding ligands are upregulated in patients with active IBD [22]–[24], suggesting that the CXCR3 axis is important in the pathogenesis of IBD [25], [26]. Although a previous study has reported attenuated DSS-induced colitis by simultaneous blockage of chemokine receptors CCR2, CCR5 and CXCR3 [27], we demonstrate that CXCR3-deficient (KO) mice alone challenged with dextran sulfate sodium (DSS) are resistant to the development of experimental colitis.

## Results

### DSS-induced colitis model

**Figure 1:**
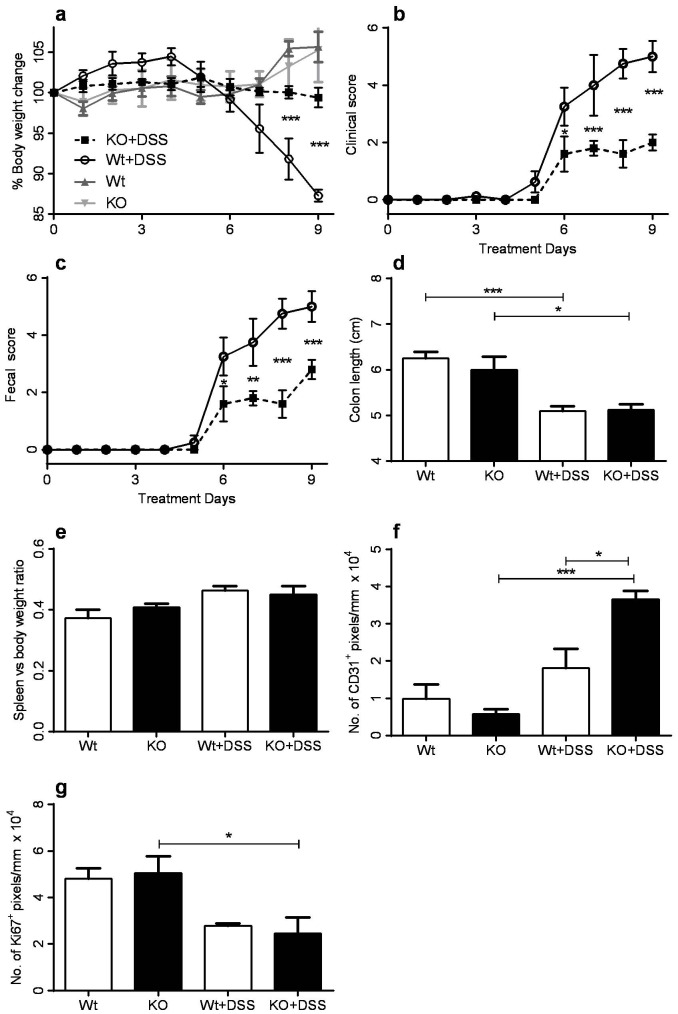
DSS-induced colitis in Wt and KO mice. **a)** Change in body weight of water- or DSS-challenged Wt and KO mice over 9 days of treatment. Data is expressed as the percentage of the original weight prior to treatment. ***p≤0.001 compared to Wt+DSS group. ***p≤0.001 compared to KO and Wt+DSS group. **b)** Corresponding clinical scores of Wt and KO mice over 9 days of DSS challenge. Clinical scores were generated by evaluating various presentations typically observed in the DSS colitis model, including reduced mobility, vocalization, and group interactions. *p≤0.05 and ***p≤0.001 compared to Wt+DSS mice. **c)** Feces of Wt and KO mice were analyzed and scored for fecal consistency, hematochezia, and rectal bleeding during the period of DSS or water challenge. **d)** Colon lengths as measured from the anus to the caecum. *p≤0.05 and ***p≤0.001. **e)** Weight of spleen on day 9 expressed as a ratio against original body weight prior to DSS or water challenge. **f)** Analysis of CD31 immunohistochemical staining of the transverse and descending colon in Wt and KO mice after 9 days of DSS challenge. **l)** Analysis of Ki67 immunohistochemical staining in the transverse and descending colon of unchallenged or DSS-challenged Wt and KO mice after 9 days. Data are represented in number of positive pixels per mm.

To examine the role of CXCR3 in the induction and development of colitis, Wt and KO mice were challenged with 2.5% DSS administered via the drinking water for 9 days. No change in normal drinking consumption was noted throughout the duration of the study in any of the treatment groups. Body weight loss, as a result of diarrhea, is a key sign in determining the severity of colitis [28]. None of the unchallenged mice (i.e., mice provided with water) developed any signs of diarrhea, rectal bleeding, or loss in body weight that are normally associated with spontaneous colitis. However, DSS-challenged Wt mice exhibited significant weight loss by day 5 and their weight continued to decline. In contrast, DSS-challenged KO mice lost less than 2% of their original weight after 9 days (Figure 1a). Overall, these data suggest that Wt mice develop more severe colitis than KO animals.

Clinical features of IBD, including mobility, grooming, group interaction, and the presence of rectal bleeding, were also monitored and scored. IBD symptoms were significantly worse in DSS-challenged Wt than KO mice from day 6 onwards (Figure 1b). Similarly, DSS-challenged KO mice also had lower fecal scores than Wt mice throughout the time course, indicative of reduced rectal bleeding, hematochezia, and loose stools (Figure 1c). Thus, the lower fecal and clinical scores, together with the reduced weight loss in DSS-challenged KO mice, are indicative of the development of less severe colitis in KO mice.

A reduction in the length of the colon is directly proportional to the severity of colitis both in patients and experimental animals [28]. However, while both DSS-challenged Wt and KO mice had shorter colons that the respective controls, there was no difference in length between DSS-challenged Wt and KO animals (Figure 1d).

The weight of the spleen also did not differ significantly between DSS-challenged Wt and KO groups. Both DSS groups had a slight increase in the overall spleen to body weight ratio when compared to water-treated animals, but this was not statistically significant (Figure 1e). This suggests that DSS-induced colitis model is not a good inducer of macroscopic systemic reactions in both Wt and KO mice.

Neovascularisation is a key pathological feature of human IBD [29], however DSS-associated neovascularisation was previously shown to be associated with repair and re-epithelialisation [30]. Consistent with this, we showed an increase in CD31^+^ staining in DSS challenged KO mice (Figure 1f), consistent with reduced histopathological scoring in this group.

IBD is also associated with abnormal cell turnover. In the colon of Wt and KO control mice, intense Ki67 staining was mainly observed in the epithelial cells, particularly at the apex of the crypts, with only mild staining in the muscular layer (not shown). However, in DSS-challenged Wt and KO mice, the 2-fold reduction in Ki67 staining (Figure 1g), and the staining pattern (not shown) reflected the significant epithelial cell loss and crypt fallout due to colitis. Significant difference was noted between KO and KO+DSS mice, however no difference was observed between DSS-challenged Wt and KO mice.

### Histopathological analysis

**Figure 2:**
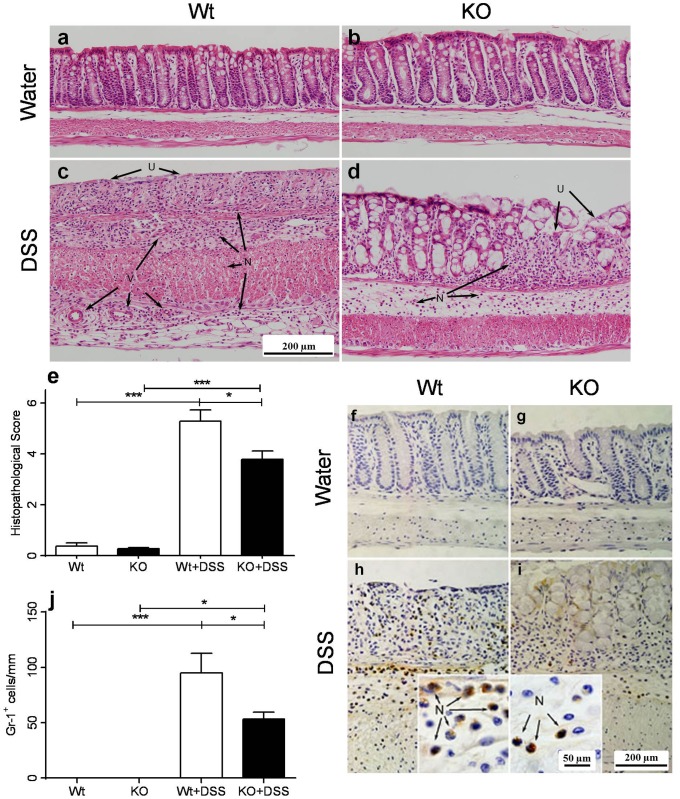
Histopathological analysis and neutrophil enumeration of the colon in Wt and KO mice after 9 days of DSS challenge. Sections of the transverse colon in **a)** control Wt mice, **b)** control KO mice, **c)** DSS-challenged Wt mice and **d)** DSS-challenged KO mice. DSS-challenged Wt mice had more cellular infiltration (N) and epithelial damage (U) than KO mice. New vascularization was observed in the submucosa and serosa layers (V). DSS-challenged KO mice exhibited less pathology as seen by relatively intact epithelium (U) and reduced cellular infiltration (N). **e)** Histopathological scores averaged from the transverse and descending colon in DSS-challenged and control Wt and KO groups. Histopathology was scored from 0 (no pathology), 5 (moderate pathology) to 10 (extreme pathology). *p≤0.05 and ***p≤0.001. **f)** Representative labelling of Gr-1^+^ cells in the **f)** control Wt mice, **g)** control KO mice, **h)** DSS-challenged Wt mice and **i)** DSS-challenged KO mice. There are sparsely scattered Gr-1^+^ neutrophils in unchallenged control Wt and KO mice. DSS-challenged Wt mice had more infiltrating neutrophils (N), particularly in the submucosa, compared to both untreated animals and DSS-challenged KO mice. **j)** Enumeration of immunohistochemically labelled Gr-1^+^ cells in the colon of both untreated animals and DSS-challenged mice. Data are represented as the number of cells per mm. *p≤0.05 and ***p≤0.001.

DSS-challenged KO mice showed sparsely distributed pathological focal points in the transverse and descending colon, whereas pathological focal points in Wt counterparts were more numerous and also more locally extensive. Furthermore, the extent of crypt damage and cellular infiltration (Figure 2a–d), as well as hematochezia (not shown), was noticeably lower in DSS-treated KO mice. Also, the epithelium was observed to be relatively intact compared to Wt counterparts. Note that the ascending colon shows little or no involvement in the DSS model of colitis and was thus not included in this study. Overall, the histopathological scores suggest that KO mice develop less severe colitis (Figure 2e).

Cellular infiltrates, such as neutrophils also contribute to IBD [28]. In unchallenged Wt and KO mice, Gr-1^+^ cells (mainly neutrophils) were sparsely scattered throughout the colon (Figure 2f and g). However, after 9 days of DSS challenge, Gr-1^+^ cells localized mainly to the submucosal region, and, to a lesser extent, in the lamina propria. These Gr-1^+^ cells tended to be associated with areas of severe ulceration and extensive pathology (Figure 2h and i). While the number of Gr-1^+^ cells increased significantly following DSS challenge in both Wt and KO mice, the number of Gr-1^+^ cells in Wt mice were still 2-fold higher than in KO mice at day 9 post-treatment (Figure 2j).

Gr-1 has recently been recognized as a marker for both neutrophils and monocytes [31]. As a result, we performed IHC labeling of Ly6C and Ly6G, specific markers for both monocytes and neutrophils, respectively. Similar to Gr-1, Ly6C^+^ and Ly6G^+^ labeling was significantly decreased (*p* = 0.0165 & *p* = 0.0018, respectively) in DSS challenged KO mice (Data S5 and S6 in File S1), suggesting abrogated chemotaxis of both neutrophils and monocytes in CXCR3 deficient mice.

### Cytokine expression in colon

**Figure 3:**
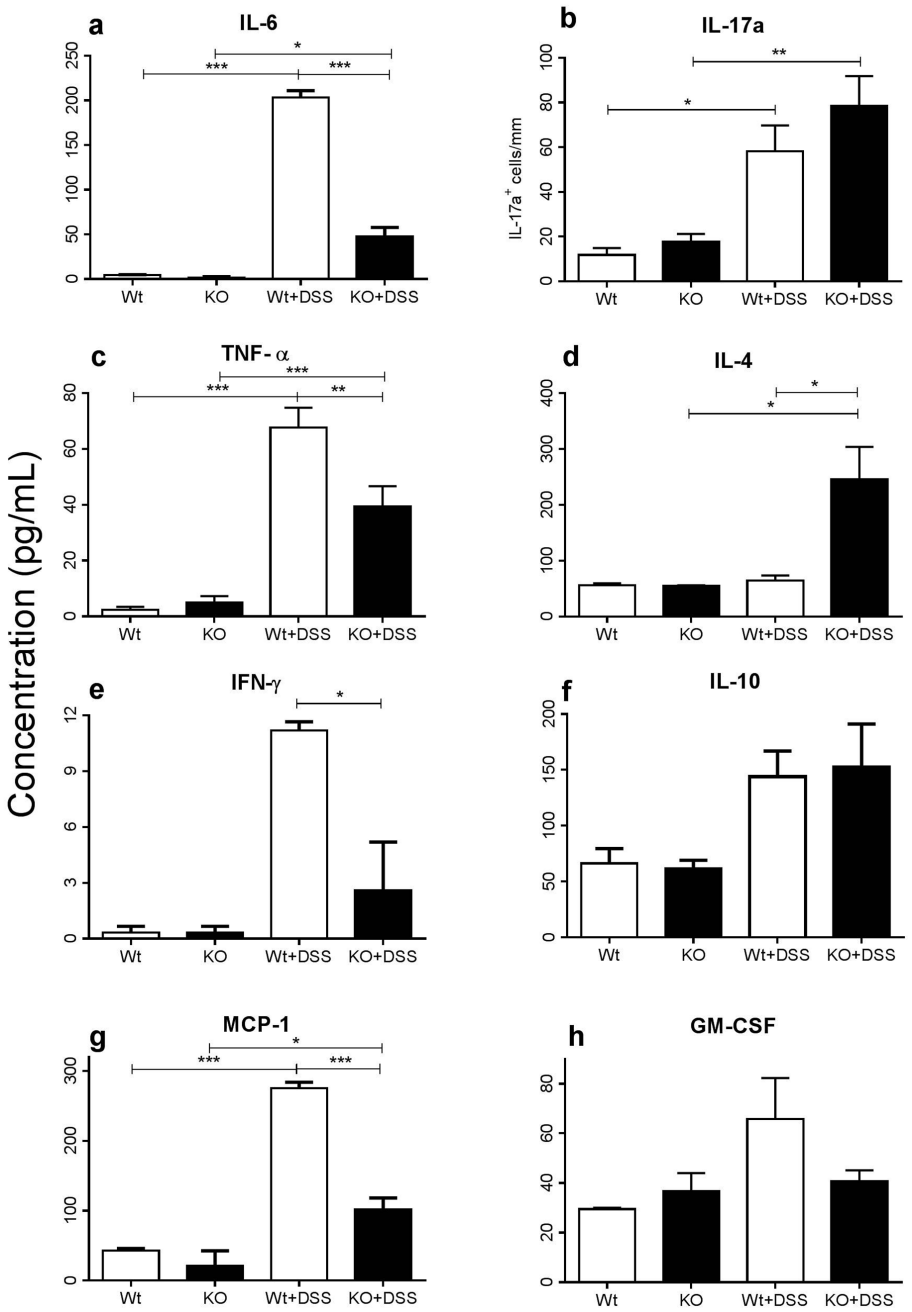
Cytokine and chemokine expression in the colon following 9 days of DSS challenge. **a)** IL-6, **b)** TNF-α, **c)** IFN-γ, **d)** MCP-1, **e)** IL-17a, **f)** IL-4, **g)** IL-10 and **h)** GM-CSF concentrations/cell counts in the colon of untreated and DSS-challenged Wt and KO mice after 9 days. Values are standardized against the protein concentration of colon homogenate supernatants. Assay was performed once and data are represented in pg/ml; *p≤0.05, **p≤0.01 and ***p≤0.001.

Proinflammatory cytokines and related factors play an important role in the pathogenesis of colitis [28]. IL-6, TNF, IFN-γ, and MCP-1 (CCL2) levels in the colon increased significantly by day 9 post-DSS challenge in Wt mice, and although still significantly increased in most cases in the KO, concentrations were significantly lower in KO mice (Figure 3). IL-17a was significantly increased in the DSS-treated Wt [32]–[34] and KO mice, as others have demonstrated. Interestingly, no significant difference was noted between DSS-treated Wt and KO mice (Figure 3b and Data S7 in File S1). IL-4 expression only increased in DSS-challenged KO mice, while IL-10 was increased 2-fold in both DSS-challenged strains (Figure 3d and f). Although GM-CSF expression was slightly higher in DSS-challenged Wt mice, this was not significant (Figure 3h). Overall, while proinflammatory cytokines increased in KO mice following DSS challenge, this response was modest compared to its Wt counterpart, indicating that the inflammatory process is reduced in CXCR3-deficient mice challenged by DSS.

### Colonic leukocytes

**Figure 4:**
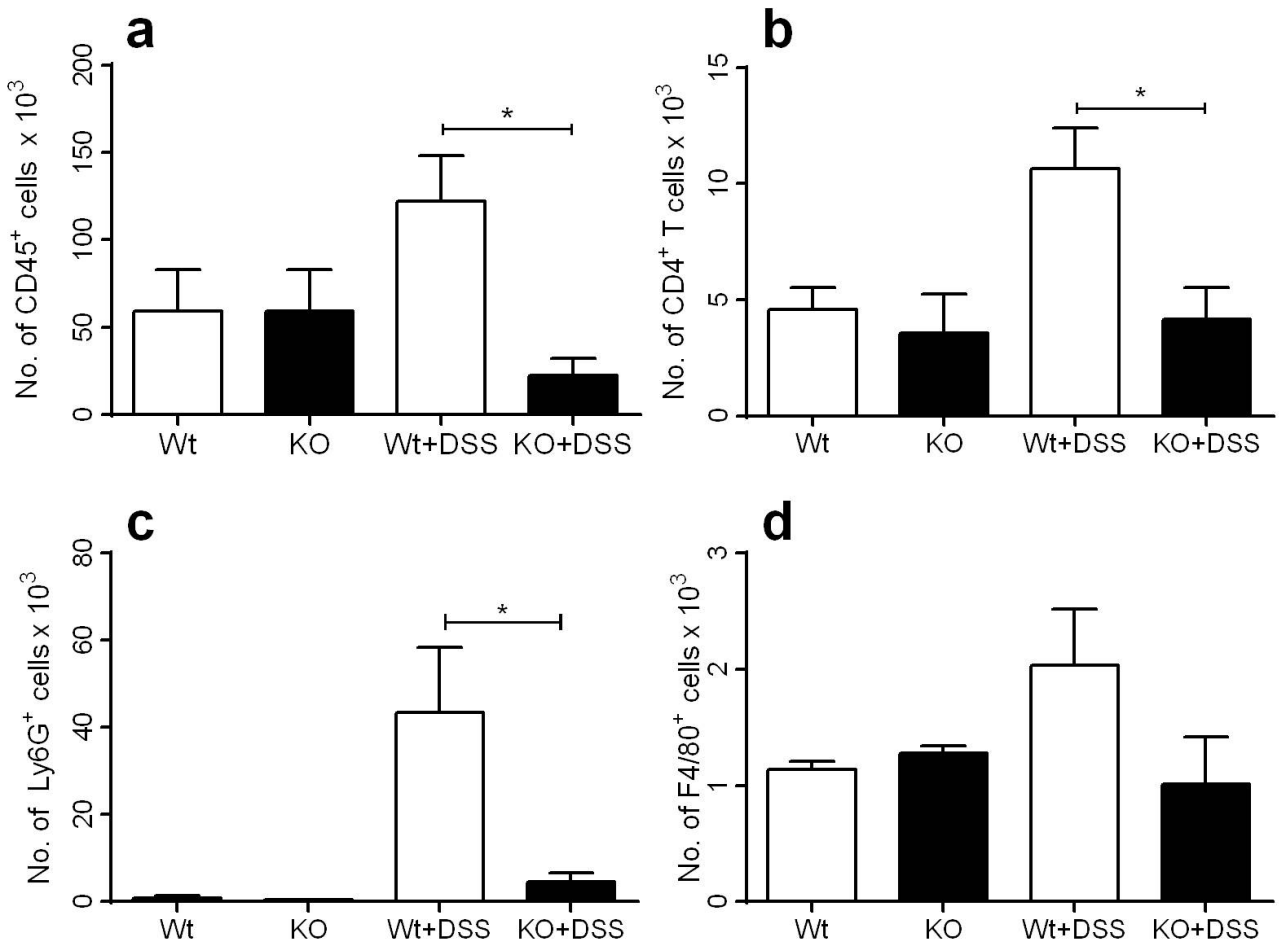
Flow cytometric analysis of leukocytes in the colon of Wt and KO mice after DSS. **a)** CD45^+^ leukocytes (CD45^+^), **b)** CD4^+^ T cells (CD45^+^ CD3^+^ CD4^+^), **c)** Ly6G^+^ neutrophils (CD45^+^ Ly6G^+^ Ly6C^+^ F4/80^−^) and **d)** F4/80^+^ macrophages (CD45^+^ F4/80^+^) in the colon following 9 days of either water or DSS-challenge. *p≤0.05.

Leukocyte infiltration mediated by CXCR3 in colitis development is not well understood. Four-fold fewer CD45^+^ leukocytes were observed in the colon of DSS-challenged KO mice compared to Wt mice despite there being similar numbers before DSS challenge (Figure 4a). More specifically, CD4^+^ T cells increased 2-fold in Wt mice by day 9 post-challenge while no change was observed in KO mice (Figure 4b). Similarly, numbers of Ly6G^+^ neutrophils only increased in DSS-challenged Wt, but not KO animals (Figure 4c). Similarly, F4/80^+^ macrophages migration appeared to decrease in DSS-challenged KO mice, albeit not significantly (Figure 4d). Taken together, these data show that in the absence of CXCR3, the migration of CD4^+^ T cells and neutrophils to the colon is impaired during colitis. Enumeration after Trypan blue staining revealed 60% cell viability, while the LIVE/DEAD Fixable Dead Cell Stain Kit showed 50% cell viability for the isolated colonic leukocytes (Data S1d in File S1).

## Discussion

Many researchers have reported on the abrogating effects of mitigated proinflammatory cell chemotaxis in the inflamed intestine. Limited influx of proinflammatory cells to the intestine in patients with IBD reduces immune-mediated secondary damage to host tissue, reducing morbidity [35]. CXCR3 and its corresponding ligands are heavily implicated in chemotaxis of proinflammtaory cells and are upregulated in patients with active IBD [22]–[24], suggesting that the CXCR3 axis is important in the pathogenesis of IBD [25], [26].

Notwithstanding the report by Tokuyama H and colleagues, showing disease mitigation with simultaneous blockade of CCR2, CCR5 and CXCR3 by TAK779 a non-peptide inhibitor [27], here, we have shown that DSS-induced colitis of CXCR3 KO mice alone resulted in markedly attenuated disease severity, as demonstrated by decreased fecal scores, clinical and histopathological signs, reduced body weight loss, and reduction in recruited proinflammatory cells and associated cytokines. Collectively, the above data suggest that CXCR3 is an important pathway in DSS-induced colitis.

CXCR3 regulates and propagates proinflammatory responses while suppressing anti-inflammatory cytokines [36]. Our results show significantly lower TNF, IFN-γ, and IL-6 levels in DSS-challenged KO mice compared to Wt animals, suggesting that an attenuated proinflammatory response is mounted. A significant increase in colonic IgG and a slight increase in IgA in DSS-challenged KO mice also may provide early evidence of a skew towards a T_h_2 response when compared to their Wt counterparts (Data, S2 and S3 in File S1). This is in line with reports implicating CXCR3 in T_h_1 responses in chronic inflammatory conditions [37], [38]. However, ELISA results of IgA and IgG in serum and intestinal wash samples did not reveal any significant increases in DSS-challenged KO mice compared to its Wt counterparts, though IgA significantly increased in DSS-challenged KO mice when compared to its water-treated KO counterpart (Data S4a-d in File S1).

Previous studies in our laboratory revealed DSS-challenged IFN-γ KO mice exhibit almost no clinical or histopathological signs of colitis, suggesting that IFN-γ may be necessary for the progression of acute experimental colitis [28]. Concordant with this, our current study revealed lower IFN-γ levels in DSS-challenged KO mice. It may be that IFN-γ-producing NK cells, which normally express CXCR3, have limited chemotaxis in CXCR3 KO mice. Although our DSS model of colitis is representative of acute inflammation, it is possible that the role of CXCR3 in chronic colitis may be mediated by IFN-γ as demonstrated in other chronic models of inflammation [39], [40], to which further investigations is required.

No statistically significant increase in IL-10 was observed in the colon from either KO or Wt mice following DSS challenge, nor was there any difference between unchallenged Wt and KO mice, suggesting that IL-10 may not be involved in DSS-induced colitis [41].

An increase in neutrophils, a key feature in the pathogenesis of colitis in humans and animals, was observed in all mice post-DSS treatment, with 2-fold higher numbers in Wt than KO animals [42]. These data suggest that CXCR3 is important in the recruitment of neutrophils to the inflammatory site following DSS challenge, in line with the findings that show that CXCR3 blockade limits neutrophil accumulation in the inflamed joints of rats with arthritis [43] and that CXCR3 is expressed by neutrophils in inflammatory sites [21], [44]. CXCL10-CXCR3 also enhances the development of neutrophil-mediated fulminant lung injury of viral and non-viral origin [44]. Neutrophils activated by IFN-γ release ROS products such as hydrogen peroxide and hypochlorous acid, causing local tissue damage [45]. Reduced levels of IFN-y in DSS-challenged KO mice suggests a reduced CXCR3-mediated neutrophil insult in the inflamed colon, potentiating the blockade of CXCR3 in reducing intestinal injury in patients with IBD.

IL-6, which also contributes to neutrophil recruitment [46], was significantly reduced in DSS-challenged KO mice compared to its Wt counterpart. However, more research is required to establish whether neutrophil migration is affected by the CXCR3 pathway, independent of IL-6. Therefore, our data suggests the importance of CXCR3 for neutrophil function at inflammatory sites.

Proinflammatory leukocyte influx and activation in the inflamed colon is pivotal in the overall pathogenesis of DSS-induced colitis and IBD [47], [48]. Accordingly, there was a 4-fold reduction in overall leukocyte numbers in DSS-challenged KO mice relative to Wt animals, with a significantly attenuated accumulation of CD4^+^ T cells and neutrophils in the colon during experimental colitis. A recent study has shown that CXCR3 is required for the generation of IFN-γ secreting T_h_1 cells *in vivo* [49], while another study has shown that IFN-γ is required for the efficient induction of CXCR3 on T_h_1 cells, thereby initiating a positive feedback loop of IFN-γ-mediated inflammation and cell trafficking [50]. As our acute DSS model of colitis is T-cell independent, the effect of reduced CD4^+^ T chemotaxis to the inflamed colon would be minimal, though a significant role is likely to be attributed in a chronic setting of inflammation.

The absence of CXCR3 resulted in significantly reduced MCP-1 levels locally, which appeared to reflect reduced macrophage numbers in the colon of DSS-treated KO mice. This suggests that particular subsets or activated cell types depend on CXCR3 for chemotaxis, thus rendering CXCR3 a promising target in IBD.

## Conclusion

Taken together, our findings demonstrate significantly attenuated DSS-induced colitis in the CXCR3 KO mice. Deficiency of CXCR3 alone has been associated with the effective reduction of colitis via reduced monocyte and neutrophil infiltration, ameliorating DSS-induced colitis, thereby making CXCR3 an attractive target for reducing inflammation. Currently, clinical trials using CXCL10 antibody (MDX-1100), are looking promising in patients with ulcerative colitis and rheumatoid arthritis [51], promoting investigations of the role of CXCR3 in chronic colitis. Our preliminary findings have shown a significant decrease in CD4^+^ T cell trafficking, as well changes in cytokine profiles that may favour T_h_2 immune responses during chronic inflammation. Future studies may help determine the role of CXCR3 in polarizing T_h_1 to T_h_2 responses in the chronically inflamed gut to evaluate the therapeutic value of targeting CXCR3.

## Materials and Methods

### Ethics Statement

All experiments were approved and carried out in according to the University of Sydney Animal Ethics Committee (AEC) (K20/9-2009/2/5146) and the Institutional Biosafety Committee (09N019) guidelines. Mice were housed in environmentally enriched AEC-approved cages. Clinical scores and body weight evaluated were performed daily to monitor disease progression and animal wellbeing. Mice that lost more than 15% of their original body weight were culled in accordance to AEC requirements. Euthansia was performed via avertin subcutaneous injection.

### Mice

Female CXCR3 KO mice on a C57BL/6 background were obtained from Iain Campbell (The University of Sydney) and were bred in the University of Sydney Blackburn Animal House along with C57BL/6 wild type (Wt) mice. Animals were provided with food and water *ad libitum*. Experiments were performed when mice were 8–10 weeks of age. All experiments were carried out 3 times (unless otherwise indicated) with 4 age-matched mice per group.

### DSS challenge

Colitis was induced in mice with 2.5% w/v DSS (ICN Biomedicals Australasia) provided *ad libitum* for 9 days. DSS induces mucosal damage in the colon, resulting in an influx of commensal flora to the submucosal layers that induces an immune response which closely parallels human colitis [28]. Control groups received tap water only.

### Body weight and clinical and faecal score

Body weight was measured twice daily and averaged [52]. Clinical scoring was based upon parameters including mobility, gait, vocalizations, group interactions, and grooming and each was scored a value between 0–2 twice daily and averaged [28]. The faecal score was based on parameters including faecal consistency, hematochezia, and rectal bleeding and measured daily [28].

### Histopathology

The colon was perfused with cold PBS, measured, and weighed. It was Swiss-rolled and sections were stained with hematoxylin and eosin as described previously [28], [53]. A histopathological score was generated in a blinded fashion using a widely used grading tool examining crypts, epithelia, goblet cells, cellular infiltration, and edema [28]. Scores of 0–1 reflect normal morphology, with 2–4, 5–7, and 8–10 representing mild, moderate, and severe colitis, respectively. Each section of colon was observed in a blinded fashion at 20x magnification and each field of view was scored as above, with the scores from 15–20 fields averaged per mouse. Only sections of transverse and descending colon were selected for histopathological assessment, as these sections clearly exhibited the greatest severity of colitis.

### Immunohistochemistry

Immunohistochemistry was performed as previously described [28]. Briefly, specimens were rehydrated, endogenous peroxidase activity blocked, and incubated either with anti-F4/80 (BM8) for 45 min or anti-Gr-1 (RB-8C5), anti-Ly6C (HK1.4), anti-Ly6G (1A8, BioLegend, CA, USA) and anti-IL-17a (Abcam, England) overnight at 4 C, or Ki67 (SP6, Abcam) for 2 h. Labeling was visualized via HRP-conjugated secondary antibody with DAB precipitate (Abcam). Positive labeling was imaged and quantified as previously described [28].

### Isolating colonic leukocytes for flow cytometry

After separation from the small intestine the colon was flushed with cold PBS and cut into 1 mm pieces before transfer into DMEM containing 1 mM dithiothreitol and 1 mM EDTA at 37°C for 1 h with shaking. The supernatant was collected and stored at 4°C. The remaining tissue pieces were treated with 0.1% collagenase IV and 0.05% DNase I (Sigma-Aldrich, MO, USA) for 30 min at 37°C. After incubation, the sample was filtered through a 70 µm nylon sieve. The supernatant was collected, pooled with the supernatant from the first incubation, and centrifuged at 250 × *g* for 5 min. The cell pellet was washed again and resuspended in 30% Percoll (Sigma-Aldrich), overlaid on 80% Percoll, and centrifuged at 250 × *g* for 20 min at 4°C with no brake. Following centrifugation, cells were aspirated and washed with FACS buffer (5% fetal calf serum, 5 mM EDTA in PBS), resuspended in FACS buffer containing anti-CD16/32 (93, 1∶100), and incubated for 30 min at 4°C. Cells were washed and incubated with a primary cocktail consisting of antibodies targeting CD45 (30-F11), CD4 (GK1.5), Ly6C, Ly6G, and F4/80 (all from Biolegend) at 1∶50 for 30 min. Unstained, isotype, and fluorescent minus one controls were included in all experiments. The LIVE/DEAD Fixable Dead Cell Stain Kit (Life Technologies, Australia) was used to determine cell viability. The samples were then washed three times, resuspended in FACS buffer, filtered through 50 µm nylon gauze, and stored at 4°C until analysis. Samples were analyzed using a FACSCanto with FACSDiva software (BD Biosciences). Acquired data was gated and analyzed with FlowJo software (TreeStar Inc. OR., USA)(Data S1 in File S1).

### Cytokine Analysis

At harvest, 0.5 cm sections of the transverse colon were collected and snap-frozen in liquid nitrogen. Samples were ground with a mortar and pestle, suspended in a cocktail protease inhibitor solution (Sigma-Aldrich), and homogenized with a rotating piston arrangement (Wheaton Specialty Glass, USA; 500 r.p.m). Samples were stored at −20°C before analysis. Cytokine analysis was performed using the BD Cytometric Bead Array kit as directed by the manufacturer (BD Biosciences).

### Statistics

All data are presented as the mean ± SEM. One-way or two-way ANOVA was performed for statistical analysis as appropriate. Differences between group means with *p* values of ≤0.05 were regarded as being statistically significantly different. Asterisks denote various *p* values as follows: * (*p*≤0.05), ** (*p*≤0.01), and *** (*p*≤0.001).

## Supporting Information

File S1 **Supporting information. Data S1)** Gating strategy used in flow cytometry experiments to determine the CD45+ leukocyte population in the colon of mice. **Data S2)** IHC-labelled IgA colonic plasma cells in unchallenged or DSS-challenged Wt and KO mice. **Data S3)** IHC-labelled IgG colonic plasma cells in unchallenged or DSS-challenged Wt and KO mice. **Data S4)** ELISA IgA and IgG measurement in serum and intestinal wash samples of unchallenged or DSS-challenged Wt and KO mice. **Data S5)** IHC-labelled Ly6C^+^ colonic monocytes in unchallenged or DSS-challenged Wt and KO mice. **Data S6)** IHC-labelled Ly6G^+^ colonic neutrophils in unchallenged or DSS-challenged Wt and KO mice. **Data S7)** IHC-labelled IL-17a^+^ colonic T cells in unchallenged or DSS-challenged Wt and KO mice.
(PDF)
