# Supplementary material for: The Role of CXCR3 in DSS-Induced Colitis
Source: PLoS One. 2014 Jul 3;9(7):e101622. doi: 10.1371/journal.pone.0101622 (PMC4081590; doi:10.1371/journal.pone.0101622)
Supplement: File S1 — Supporting information. Data S1) Gating strategy used in flow cytometry experiments to determine the CD45+ leukocyte population in the colon of mice. Data S2) IHC-labelled IgA colonic plasma cells in unchallenged or DSS-challenged Wt and KO mice. Data S3) IHC-labelled IgG colonic plasma cells in unchallenged or DSS-challenged Wt and KO mice. Data S4) ELISA IgA and IgG measurement in serum and intestinal wash samples of unchallenged or DSS-challenged Wt and KO mice. Data S5) IHC-labelled Ly6C+ colonic monocytes in unchallenged or DSS-challenged Wt and KO mice. Data S6) IHC-labelled Ly6G+ colonic neutrophils in unchallenged or DSS-challenged Wt and KO mice. Data S7) IHC-labelled IL-17a+ colonic T cells in unchallenged or DSS-challenged Wt and KO mice. (PDF) [file pone.0101622.s001.pdf]

Supplemental Figures

Data S1

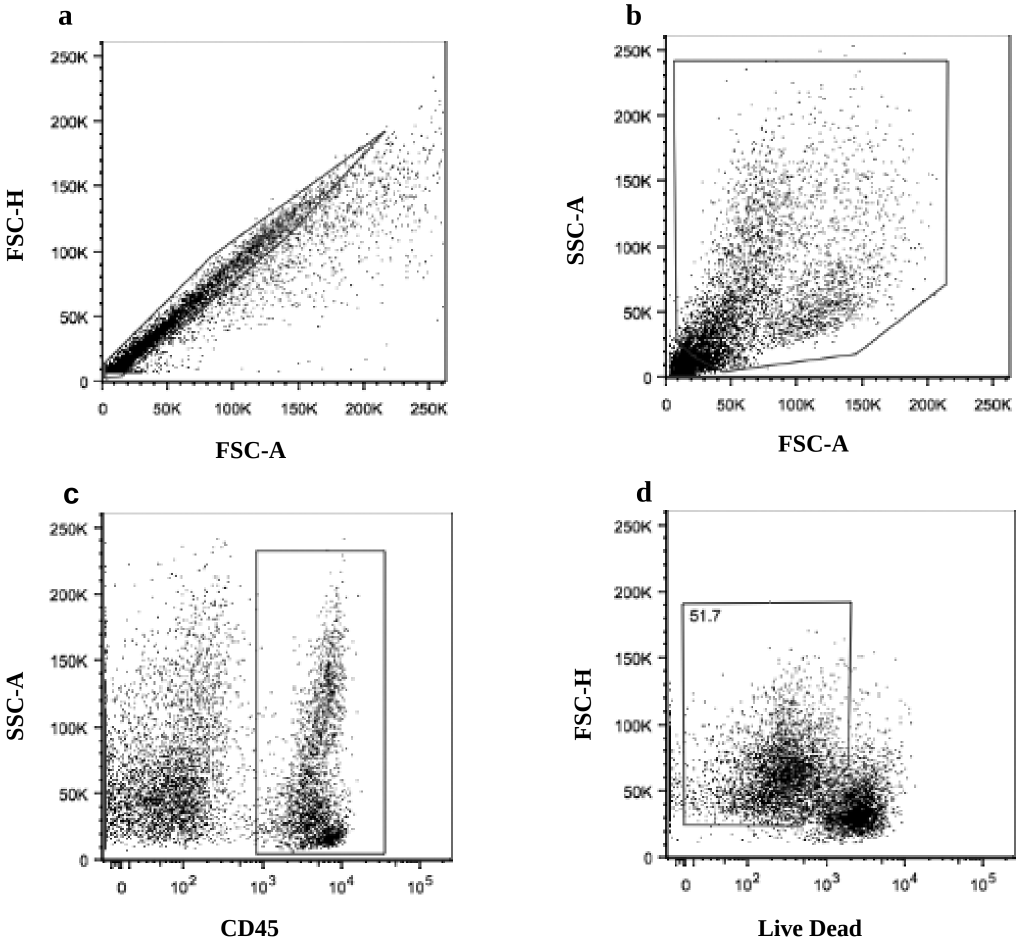

Figure S1: Gating strategy used in flow cytometry experiments to determine the CD45+ leukocyte population in the colon of mice. Representative flow cytometry dot plots showing **A)** gating for single cells isolated from the colon, **B)** gating to remove debris from the 'single cell' population, **C)** gating for leukocytes (CD45+) in the debris-free single-cell population, and **D)** gating for live cells in the CD45+ population. Analysis of cell surface markers CD45, CD4, Ly6G and F4/80 was performed after these initial gating strategies. Dot plots represent of 1 independent experiment with n=4 mice per group.

Supplemental Figures

Data S2

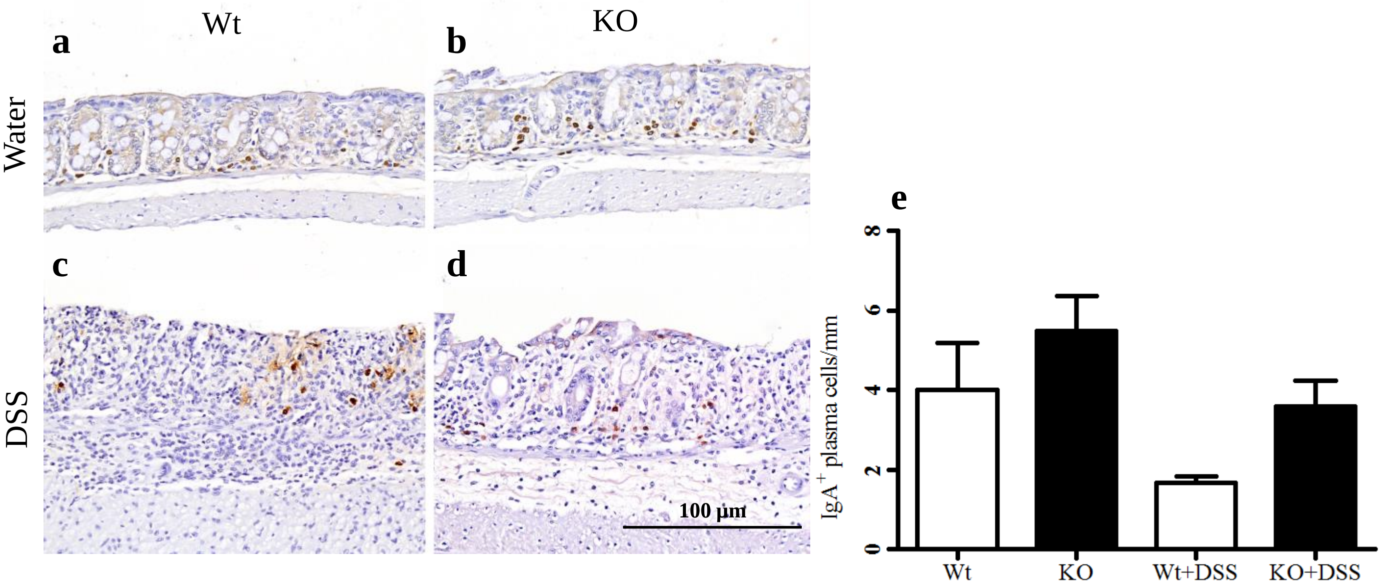

Data S2. IgA colonic plasma cells in unchallenged or DSS-challenged Wt and KO mice.

Representative labelling of IgA^+ plasma cells in water treated **a)** Wt and **b**) KO mice and DSS challenged **c)** Wt and **d**) mice, respectively. e**)** Enumeration of IgA^+ plasma cells in the colon following 9 days of either water or DSS challenge.

Data S3

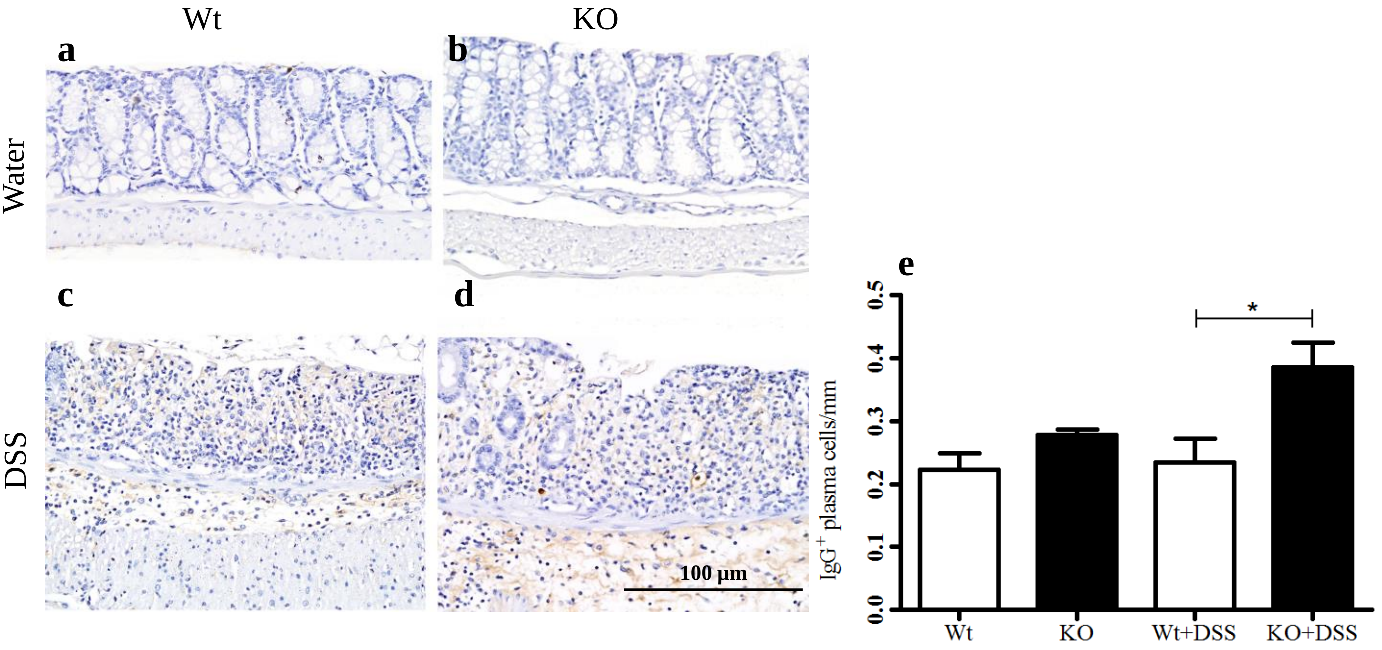

Data S3. IgG colonic plasma cells in unchallenged or DSS-challenged Wt and KO mice.

Representative labelling of IgG^+ plasma cells in water treated **a)** Wt and **b)** KO mice and DSS challenged **c)** Wt and **d)** mice, respectively. **e)** Enumeration of IgG^+ plasma cells in the colon following 9 days of either water or DSS challenge.* $p \leq 0.05$

Supplemental Figures

Data S4

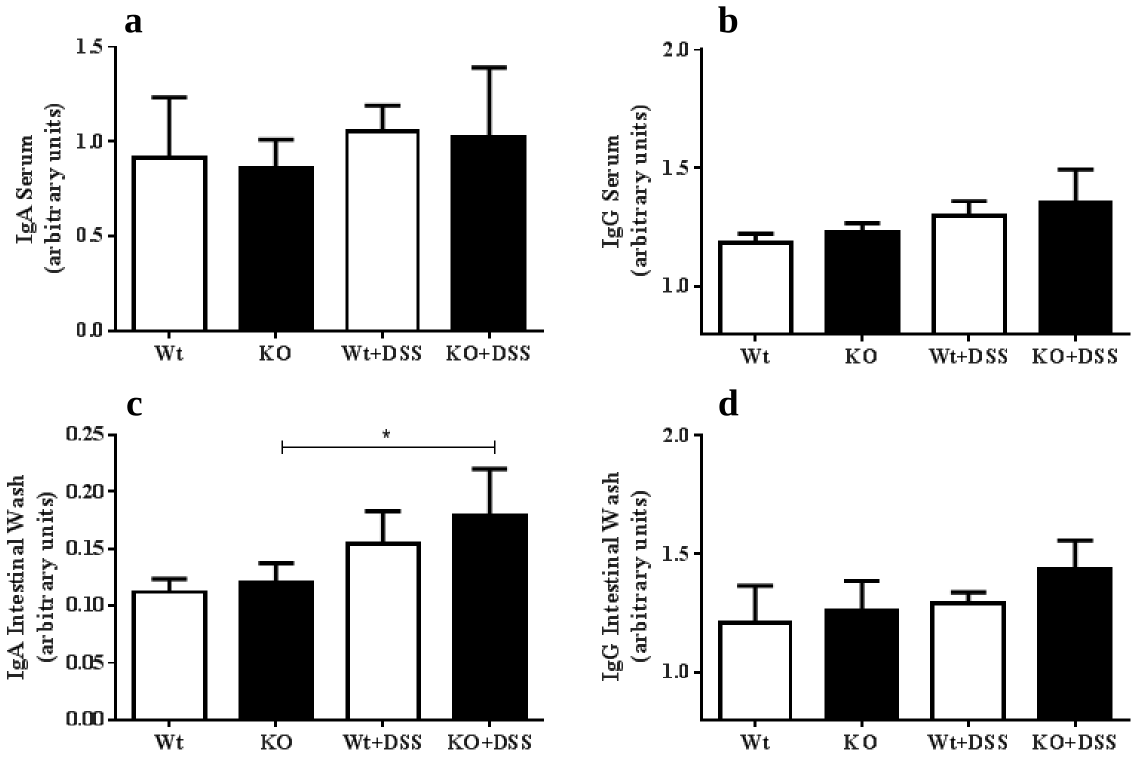

Data S4. ELISA IgA and IgG measurement in serum and intestinal wash samples of unchallenged or DSS-challenged Wt and KO mice. IgA and IgG assayed in **a-b)** serum and **c-d)** intestinal wash samples in the colon following 9 days of either water or DSS challenge, respectively.* $p \leq 0.05$. Briefly, sandwich ELISA was performed by coating plates with sheep anti-mouse Ig antibody (1:200 v/v) overnight, followed by 1% skim milk block for 1 h at 37˚C. Following this, samples were incubated at room temperature for 1 h and wash before the detection antibody, rabbit anti-mouse IgA or IgG (1:200 v/v) was added for an additional 1 h at room temperature. Following washing, anti-rabbit Ig HRP (1:200 v/v) conjugated secondary was incubated for 30 min, washed and developed with ABTS for 15-30 min. 1% SDS solution stop solution was added to the plate and the absorbance was read with an spectrophotometer at 410nm. Data is presented in arbitrary units.

Supplemental Figures

Data S5

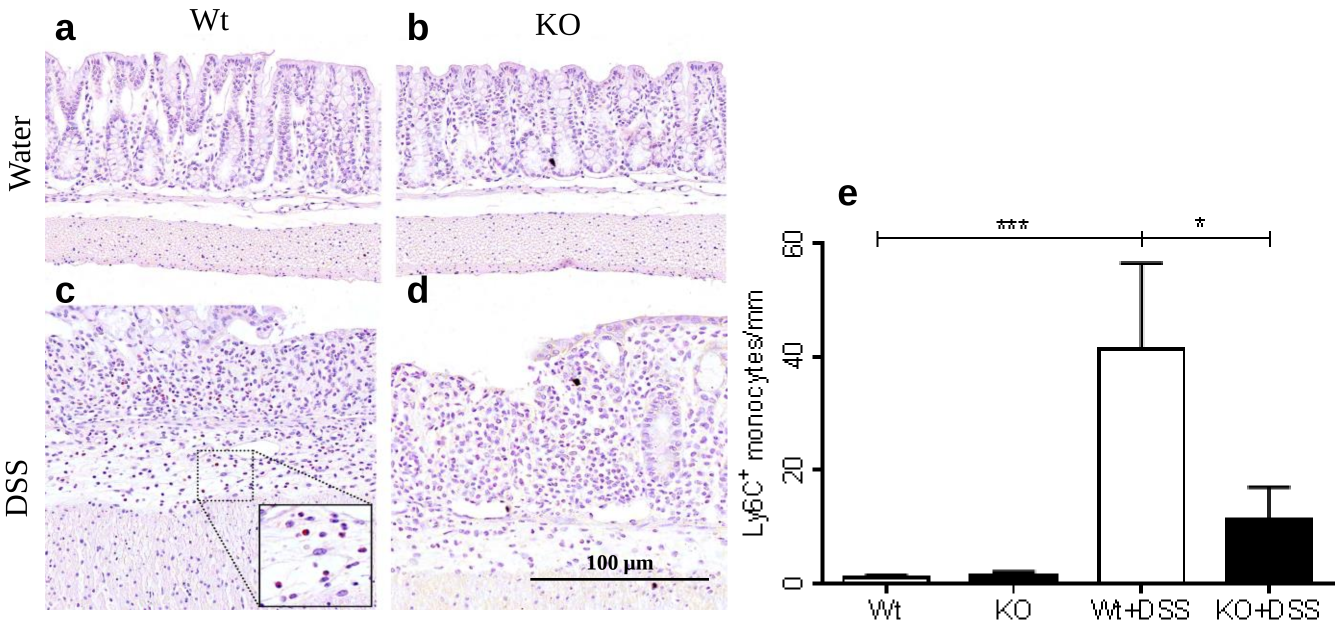

Data S5. Ly6C^{+} colonic monocytes in unchalle

Representative labelling of Ly6C^{+} cells in water treated **a)** Wt and **b**) KO mice and DSS challenged **c)** Wt and **d**) KO mice, respectively. e**)** Enumeration of Ly6C^{+} cells in the colon following 9 days of either water or DSS challenge.* $p \leq 0.05$ & *** $p \leq 0.0001$

Data S6

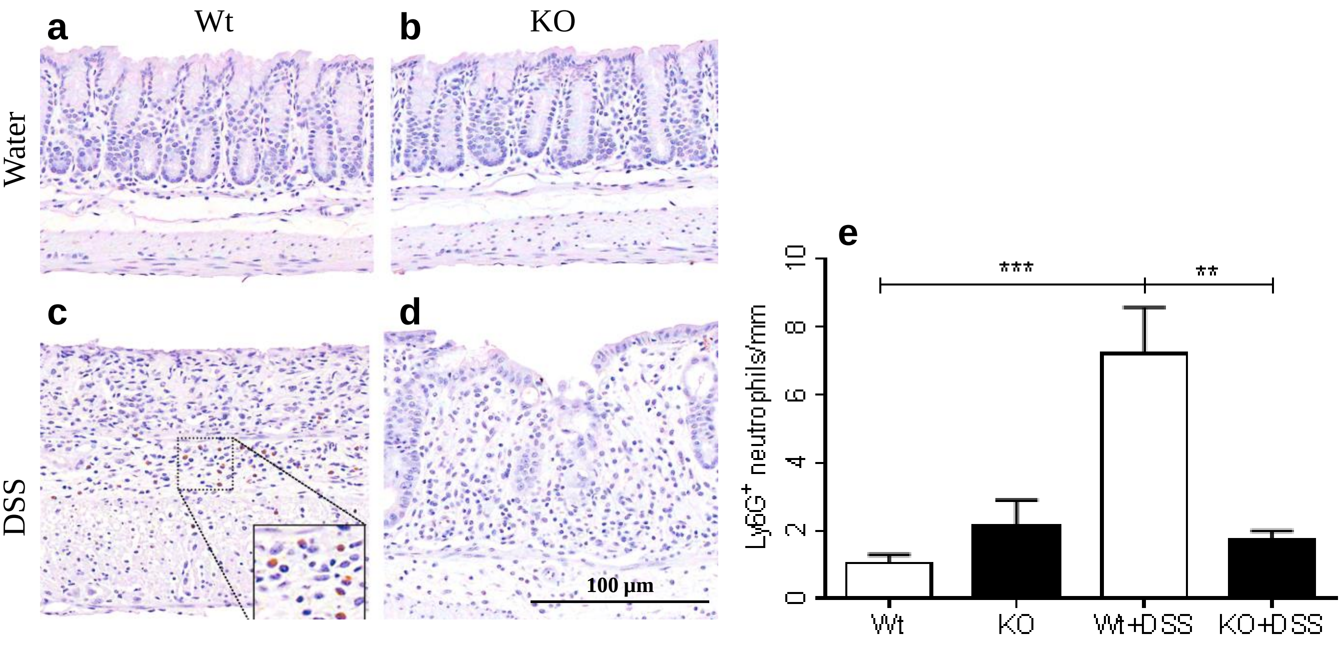

Data S6. Ly6G^{+} colonic neutrophils in unchallenged or DSS-challenged Wt and KO mice. a) Representative labelling of Ly6G^{+} cells in water treated **a)** Wt and **b**) KO mice and DSS challenged **c)** Wt and **d**) KO mice, respectively. e**)** Enumeration of Ly6G^{+} cells in the colon following 9 days of either water or DSS challenge.* *$p \leq 0.001$ & *** $p \leq 0.0001$

Supplemental Figures

Data S7

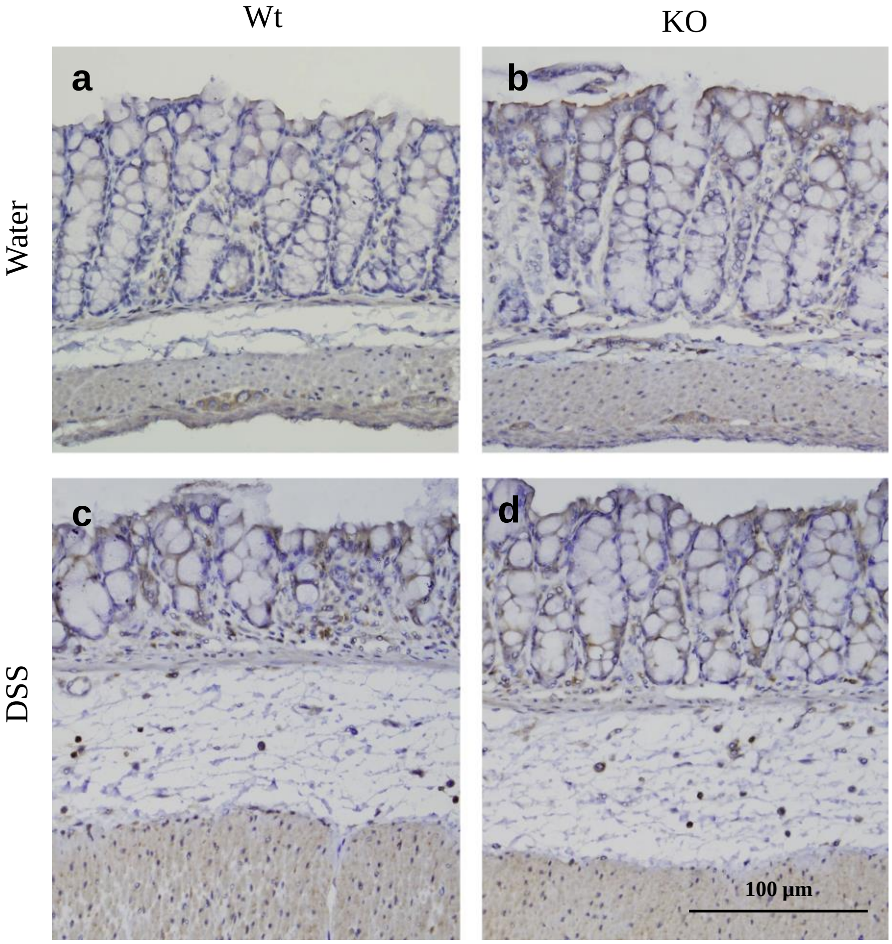

Data S7. IL-17a^{+} colonic T cells in unchallenged or DSS-challenged Wt and KO mice.

Representative labelling of IL-17a^{+} cells in the submucosa of water treated **a)** Wt and **b**) KO mice and DSS challenged **c)** Wt and **d**) KO mice, respectively.
